# On the Efficiency of a Piezoelectric Energy Harvester under Combined Aeroelastic and Base Excitation

**DOI:** 10.3390/mi12080962

**Published:** 2021-08-14

**Authors:** Antiopi-Malvina Stamatellou, Anestis I. Kalfas

**Affiliations:** Laboratory of Fluid Mechanics and Turbomachinery, Mechanical Engineering Department, Aristotle University of Thessaloniki, 54124 Thessaloniki, Greece; akalfas@auth.gr

**Keywords:** energy harvesting, piezoelectric transducers, aeroelasticity, base vibration

## Abstract

A flutter-type, nonlinear piezoelectric energy harvester was tested in various combinations of aerodynamic and harmonic base excitation to study its power output and efficiency. The commercial polyvinylidene fluoride film transducer LDT1-028K was used in 33 excitation mode. The aerodynamic excitation was created by a centrifugal fan and the base excitation by a cone speaker. The excitations were produced by varying independently the mean airflow velocity and the frequency of base vibration. A capacitive load was used to store the harvested energy. A line laser was employed along with long exposure photography and high-speed video, for the visualization of the piezo film’s mode shapes and the measurement of maximum tip deflection. The harvested power was mapped along with the maximum tip deflection of the piezo-film, and a process of optimally combining the two excitation sources for maximum power harvesting is demonstrated. The energy conversion efficiency is defined by means of electrical power output divided by the elastic strain energy rate of change during oscillations. The efficiency was mapped and correlated with resonance conditions and results from other studies. It was observed that the conversion efficiency is related to the phase difference between excitation and response and tends to decrease as the excitation frequency rises.

## 1. Introduction

The exploitation of ambient energy, producing electric power in the order of milliWatts is called energy harvesting. The application can be useful in powering low power electronics, reducing the need for battery replacement, maintenance, and recharging. Ambient energy is available in abundance in nature and the industry, where there is also a need for widely distributed, self-powered sensor networks that ensure optimal operation of components since wiring and installation costs account for more than half of the total cost of distributed sensor measurement systems. In this direction, energy harvesting further enables the installation of more sensors and the placement of sensors in places where wiring is not feasible. The design of wireless sensor network (WSN) nodes powered by piezoelectric energy harvesters (PEHs) is currently considered for practical application with temperature sensing being a relevant application [1].

Today, most wireless measuring devices and networks need frequent battery replacements that limit the extension of their use. Energy harvesting technologies present a viable solution that will decrease the need for these replacements. Energy harvesters can complementarily power wireless devices and decrease the number of times battery replacement is needed. In the last decade, studies have been concerned with thermoelectric, piezoelectric, electromagnetic, and photovoltaic energy harvesting [2]. As regards industrial applications, ABB has successfully introduced the TSP300-W, the first wireless temperature sensor [3] powered by a thermoelectric energy harvester along with a battery. As another example, TDK introduced its “InWheelSense” piezoelectric energy harvesting and sensing module [4] that generates power from vehicle wheel vibrations while also serving as a sensing platform to enable a variety of vehicle data-collection functions.

Piezoelectric energy harvesting (PEH) is the most popular energy-harvesting concept exploiting ambient kinetic energy through the vibration of piezoelectric transducers. Kinetic energy exists in many applications of the built environment such as engines, heavy equipment, highways, and railways [5]. Autonomous condition monitoring of rotating machinery is a possible application of PEHs [6,7], as well as health monitoring of aerospace structures [8]. The field of PEH evolved from the field of passive vibration damping [9], which employs mechanical systems or fluids to reduce vibration in machinery. The main disadvantage of PEHs is their limited power output when the excitation frequency is far from the transducer’s resonance frequency and efforts are made to broaden their operation bandwidth [10,11,12].

Piezoelectric transducers can be excited from their base vibration and also with flow-induced excitation. These two excitation modes often coexist in nature and industrial environments. For example, a PEH transducer installed on a wind turbine’s blade is subjected to flow excitation from surrounding air and base excitation from the blade’s vibration. Additionally, a PEH transducer installed on an HVAC duct is excited by the airflow in the duct and vibrating with the duct’s wall [13]. PEHs tend to vibrate on their eigenfrequency when subjected to airflow [14]. Flutter-type PEHs are elastic films clamped at their leading edge and free at their trailing edge. The flow excitation has been realized by Karman vortex street created by bluff bodies in water [15] and air [16]. The Karman vortex street has been extensively used in experiments because of the predictability of the flow excitation it creates. Harvesters employing new concepts have been presented such as the use of sliding bluff bodies to increase the power output [17], nested bluff-body structures in tandem arrangements [18], and coupling of vortex-induced vibration and galloping phenomena [19,20]. The differences in bluff body shapes were found to bring distinct dynamic behaviors [20].

A variation of the flutter-type harvester is the inverted piezoelectric flag [21] with which an application of autonomous wind speed measurement was presented [22]. The inverted flag is an orthogonal film with a free leading edge and a fixed trailing edge. It has been proposed that the vibration of an inverted flag can be predicted with the use of the nondimensional bending stiffness, β [21]. The parameter β compares the magnitude of the bending force with the fluid inertial force.

(1) $$\beta =\frac{Ε\cdot h^{3}}{12\cdot \rho_{f}\cdot U^{2}\cdot L^{3}\left(1-v^{2}\right)}$$

where *E* is Young’s modulus, $\rho_{f}$ is the fluid density, *ν* is Poisson’s ratio, *U* is the mean flow velocity, *h* is the thickness and *L* is the length of the flag. If *β* is in the range 0.1≤β≤0.3 then the flag has a sustained flapping [21]. Flapping is the desired state for the piezoelectric film in PEH as it increases the acting stresses and thus the output energy.

Piezoelectric energy harvesters are excited through contact with vibrating sources and with flow-induced vibration. The effect of a combined excitation has only recently started to be studied [23,24,25]. The aeroelastic excitation has been created by vortex-induced vibration, flutter, and galloping phenomena. In all cases, the models are supported by experimental validation, which allows a better insight into the harvester’s behavior. A previous paper by the authors [14] investigated two piezo film transducer types, placed on a specially devised experimental setup with combined excitation. The study brought insight into the frequency range with the maximum power output for piezo-film transducers by analyzing voltage output measurements.

The “general 1-D model of piezoelectric vibration energy” with a base excitation has been presented by du Toit [26]. This model was extended to account for the combination of aeroelastic and base excitation of the transducer, as described by the following electromechanically coupled equations:

(2) $$\ddot{w}+2\zeta_{m}\omega_{Ν}\dot{w}+\omega_{N}{}^{2}w-\omega_{Ν}{}^{2}d_{33}v=-\ddot{w_{B}}-\frac{F_{flow}}{m_{eff}}\;$$

(3) $$R_{eq}C_{p}\dot{v}+v+m_{eff}R_{eq}d_{33}\omega_{Ν}{}^{2}\dot{w}=0\;$$

where *w* is the harvester’s displacement, *w_B_* is the base displacement, *v* is the voltage, *ζ_m_* is the mechanical damping ratio, and *ω_N_* is the natural frequency of the device. The piezoelectric element has a mass *m_p_* and is connected to a power-harvesting circuit, modeled as a resistor. *M* is a proof mass placed at the transducer’s tip. The approximate effective mass is *m_eff_ = M + m_p_*, and d_33_ is the piezoelectric constant relating charge density to stress. The 33 excitation mode is used in these equations. *F_flow_* is added as an average, fluctuating aerodynamic force acting on the piezoelectric element to simply describe the aerodynamic force. The 1-D model is sufficient for a first approximation of the piezo beam’s vibration. However, its linear character does not allow it to be applied in flutter-type piezo-film oscillations with a high amplitude, which are strongly nonlinear.

An important parameter for energy harvesters is the energy conversion efficiency that has been defined with various formulae in the literature. The efficiency is critical for the design optimization of PEHs and the comparison of PEHs with alternative power generation systems (thermal energy harvesters, nanogenerators, etc.). The current study proposes a definition of energy conversion efficiency, which covers only a part of the energy flow in the harvesting system, explained as follows: If one observes the energy flow in an energy-harvesting system, three parts are essential—the excitation source (e.g., structural vibration), the energy-harvesting device (e.g., PEH), and the interface circuit to the electrical energy storage (e.g., capacitor or battery). The following energy transformation steps may be observed: (i) mechanical energy is transferred from the excitation source to the PEH structure, where it is converted to strain energy; (ii) strain energy is converted to electric charge; (iii) the electrical energy is extracted from the PEH by means of a rectifier circuit and transferred to the external load, capacitor, or battery.

Energy conversion efficiency is defined as the ratio of the output energy to the input energy of a system. For PEH, this is the ratio between the output electrical energy and the input mechanical energy. As discussed in [27], this definition has some similarities to the electromechanical coupling factor squared $k_{ij}{}^{2}$, where $k_{ij}$ is the material coupling factor (electrical field in direction *i*, stress in direction *j*). This parameter is reported by the suppliers of piezoelectric material, to show the efficiency of the piezoelectric material in converting strain energy into electrical energy, not taking into account the specific structural design and rectification circuit. The overall harvester efficiency is usually much smaller than the material coupling factor $k_{ij}$. The concept of coupling factor, or the coupling coefficient, has also been defined at the system level [28] for the piezoelectric structure as

(4) $$\;k_{sys}=\frac{\left(\omega_{o}^{2}-\omega_{s}^{2}\right)}{\omega_{o}^{2}}$$

where *ω_o_* and *ω_s_* are the open-circuit and short-circuit natural frequencies of the vibration mode of interest. It is a measure of mechanical to electrical energy conversion within the lossless structure, and it does not account for the effects of the electrical load or the mechanical and dielectric losses.

Various expressions for the input and output energy have been proposed, and this is a reason for the large discrepancy in efficiency values reported. In 2006, Shu and Lien [3] theoretically analyzed the energy conversion efficiency of a cantilever PEH, coupled with a full-bridge rectifier around resonance states. They assumed that the input mechanical energy was the sum of extracted electrical energy and the energy dissipated by the structural damping. The efficiency expression was

(5) $$\eta =\frac{\alpha \kappa^{2}}{\zeta {\left(\alpha \tilde{\omega }+\pi /2\right)}^{2}+\alpha \kappa^{2}}$$

According to this definition, efficiency depends on the frequency ratio $\tilde{\omega }$ (response frequency/natural frequency), the normalized resistance α, the electromechanical coupling coefficient $\kappa^{2}$ and the mechanical damping ratio ζ. This definition indicates that efficiency increases with a large coupling coefficient and a small damping ratio. Additionally, it is known that due to the stiffness difference of piezoelectric elements between the short-circuit (*ω_n_* = 1) and open circuit condition (ω_n_ $=\sqrt{1+\kappa^{2}}$), the frequency to obtain the maximum power output shifts with the variation of the coupling factor [26]. As expected, a higher coupling coefficient is associated with higher PEH efficiency. Since the coupling coefficient is inversely proportional to the bending stiffness and the internal piezoelectric capacitance, designs with thin films and small internal capacitance are proven more efficient harvesters.

Several experimental studies have been conducted that investigate the capability of PEHs to extract energy. However, most lack an energy-saving circuit, and their findings on the expected power output are vast estimations. In this study, real-time measurements of the output power were performed with a piezoelectric film transducer excited with both aerodynamic and base excitation. This offers a realistic measurement of the harvested energy. The piezoelectric transducer LDT1-028K was used because of its performance during previous studies [14], and an experimental setup was devised in order to provide improved aerodynamic and base excitation for the specific transducer. The aerodynamic excitation of the harvester was chosen using findings from previously used experimental setups [29].

The study of the power output of a piezoelectric harvester with large deflections, in regard to its mode shapes, was attempted, using laser visualization. Additionally, power output measurements and capacitor charging curves from combined excitation are presented, and conversion efficiency was defined, measured, and mapped in the combined excitation space. The parameters that influence a flutter-type piezo film’s power output are under research and include the base vibration frequency, the film’s vibration frequency, and its tip deflection and curvature. There are always uncertainties associated with the mathematical modeling, which result from the numerous assumptions made when modeling the geometry, the boundary conditions, and the behavior of the materials [30]. Identification of the important parameters of the physical problem through experiments is a necessary step for accurate modeling and the findings of the study are useful for validation and improvement of existing PEH models. The results of this study are of practical importance for wireless sensor networks used in structural health monitoring and other applications.

## 2. Materials and Methods

The piezoelectric energy-harvesting system consisted of two excitation sources—the piezoelectric transducer and the rectification and energy storage circuit. The two exciters were a 120 W centrifugal fan that created a controlled aerodynamic excitation and a cone speaker that created a sinusoidal base excitation. Experiments for this study were carried out in a 700 × 600 × 300 mm^3^ space in the Laboratory of Fluid Mechanics and Turbomachinery at the Aristotle University of Thessaloniki.

The commercial piezoelectric film transducer LDT1-028K was used. The film consists of a layer of fluoropolymer polyvinylidene fluoride (PVDF) covered in two sheets of polyester film. Piezopolymer film has been incorporated into sensing, actuation, and energy harvesting due to its flexible nature and biodegradability [31]. Having one active piezoelectric layer LDT1-028K is a unimorph. The film’s characteristics are presented in Table 1. The power output of the specific transducers in energy-harvesting applications is usually of the order of 1 μW [32,33], while low-power electronics need power in the order of 1 mW to function.

PVDF and other piezoelectric polymers are used in energy harvesting of vibrational energy because of their flexibility and low weight in comparison to piezoceramics that have higher piezoelectric charge coefficients, yet are brittle. PVDF can also withstand vibration fatigue. The operating temperature of LDT1-028K is 0 °C to 70 °C and the modulus of elasticity is in the range from 2 to 4 GPa as stated by the manufacturer. The natural frequency of the piezo film has been measured with an impact test [13].

The direct piezoelectric effect is described by the linear theory of piezoelectricity [34] by the following relation:

(6) $$D_{i}=d_{ij}\cdot T_{j}+\varepsilon_{ik}^{T}\cdot E_{k}$$

where *D_i_* is the dielectric displacement [C/m^2^], *d_ij_* is the piezoelectric charge coefficient [Cb/N], and *T*_j_ is the applied stress [N/m^2^]. At *d_ij_*, the first subscript is the direction of the charge motion associated with the applied stress, while the second subscript is the direction of mechanical stress. Moreover, *E_k_* is the electric field [V/m] and k its direction, and *ε_ik_* is the piezoelectric constant [F/m] tensor under constant stress.

Coefficient *d_ij_* quantifies the charge output of a piezoelectric material when stress is applied in a given direction. The coefficient is given as a third-order tensor that is expressed as a 3 × 6 matrix that correlates the charge displaced unit area associated with applied stress. For an unstretched and poled PVDF polymer the matrix form can be written as follows:

(7) $$\left[\begin{array}{c} D_{1}\\ D_{2}\\ D_{3} \end{array}\right]=\left[\begin{array}{ccc} 0 & 0 & 0\\ 0 & 0 & 0\\ d_{31} & d_{32} & d_{33} \end{array}\;\;\;\;\begin{array}{ccc} 0 & d_{15} & 0\\ d_{24} & 0 & 0\\ 0 & 0 & 0 \end{array}\right]\cdot \left[\begin{array}{c} \begin{array}{c} T_{1}\\ T_{2}\\ T_{3} \end{array}\\ T_{4}\\ T_{5}\\ T_{6} \end{array}\right]+\left[\begin{array}{ccc} \varepsilon_{11}^{Τ} & 0 & 0\\ 0 & \varepsilon_{22}^{Τ} & 0\\ 0 & 0 & \varepsilon_{33}^{Τ} \end{array}\right]\cdot \left[\begin{array}{c} E_{1}\\ E_{2}\\ E_{3} \end{array}\right]$$

**Figure 1:**
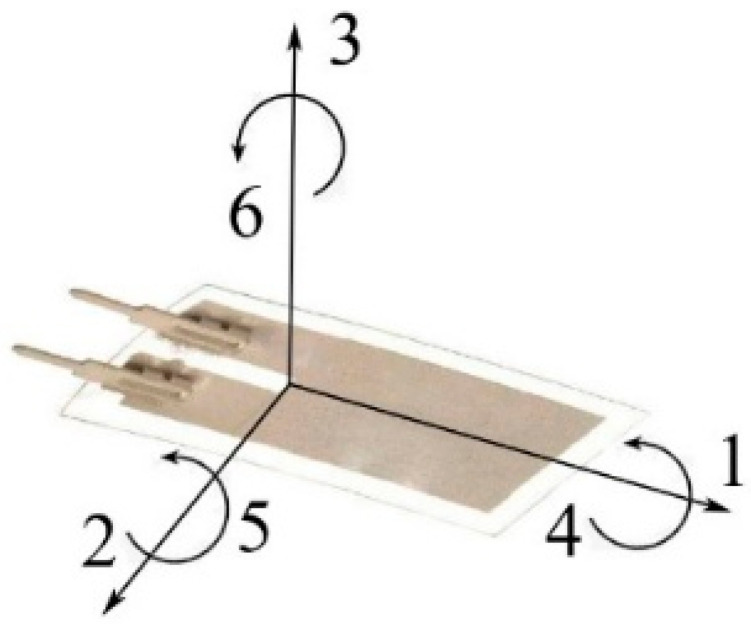
Stress directions for the piezoelectric film.

Polarization of the PVDF film is performed in direction 3 as the electrodes are placed on the surface of the film in the manufacturing phase [35]. Stresses 1–3 are normal and 4–6 are shear stresses. The stress direction symbols are presented in Figure 1.

As the piezo film is used as a flutter-type harvester, it is subjected to bending, and the stress is mainly in direction 1. The values of the piezoelectric charge coefficients are given in Table 2.

**Figure 2:**
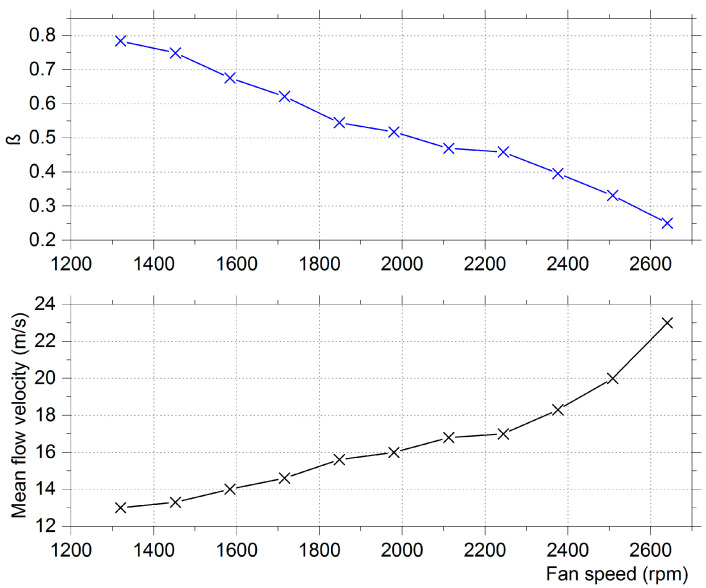
Dimensionless bending stiffness (β) and mean airflow velocity as functions of the fan’s speed.

The fan’s maximum speed was 2640 rpm. Its operation curve is presented in Figure 2. The mean flow velocity measurements were taken with a hot film anemometer. For each measurement point, 3 measurements were taken across the vertical diameter of the fan’s exit. The piezoelectric film’s tip was at a 40 mm distance from the fan’s exit throughout the experiments. The fan’s exit had a Ø86 mm circular cross section. The pressure fluctuations acted as excitation for the piezoelectric film. The fan speed was varied by an inverter from 1320 to 2640 rpm. For the specific setup, the dimensionless bending stiffness computed according to Equation (1) was in the range of 0.25–0.78, as seen in the diagram of Figure 2. The configuration used in this study is a flutter-type harvester with a fixed leading edge and a free trailing edge. The flow did not present large amplitude vibrations on the piezo-film maximum, and thus, the mean flow velocity was below the flutter speed.

**Figure 3:**
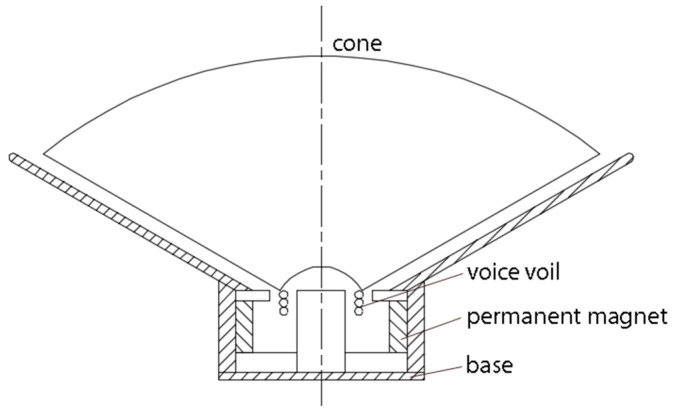
Drawing with the cone speaker’s main parts.

A sinusoidal excitation of the piezoelectric energy harvesters’ base is frequently present in computational studies with PEHs [38,39]. This base excitation was experimentally realized using a commercial cone speaker (50 W rms, 4 Ω impedance, and 180 mm diameter) that converted the input electrical signal into oscillating motion. The sinusoidal input signal was created by a signal generator (RIGOL DG1022) and was then amplified by two amplifiers, a vacuum tube preamplifier and a 2 × 25 W rms main amplifier. The input signal had a consistent peak-to-peak voltage and its frequency varied. The cone speaker’s parts are presented in the drawing of Figure 3.

**Figure 4:**
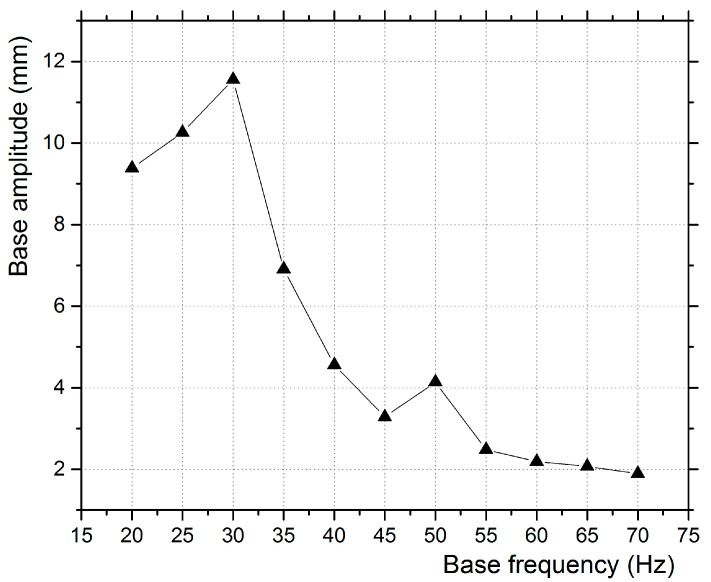
Frequency vs. amplitude diagram of the cone speaker.

**Figure 5:**
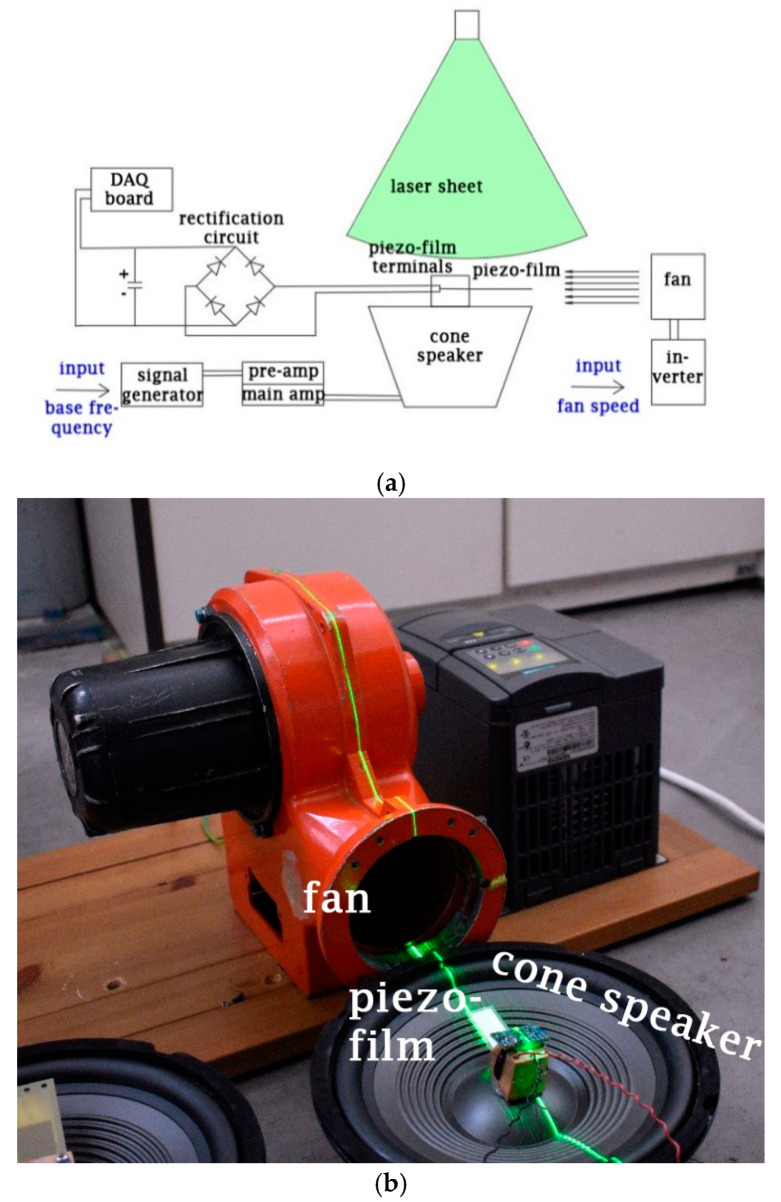
(**a**) Schematic representation of the experimental setup; (**b**) detail of the setup showing the relative position of the fan with respect to the piezo film and the laser beam position. The piezo film was mounted on the dust cap of the cone speaker.

The cone was connected to a fixed coil that served as a temporary magnet, placed in front of a permanent magnet. The input electric signal was translated by the alternating magnetic force to an oscillating motion of the cone known as an excursion. The cone speaker’s vibration amplitude varied throughout the vibration frequency range, as shown in Figure 4. The piezoelectric film was mounted on a wooden base in the center of the cone speaker, as seen in Figure 5, in a horizontal direction with zero incidence angle with respect to the average incoming flow velocity. The maximum amplitude of the excitation was observed at 30 Hz, near the cone’s natural frequency. The cone speaker produced a sinusoidal response to the sinusoidal input signals that can be described as follows:

(8) $$z_{B}=z_{0}\cos\left(\omega \cdot t\right)$$

where *z_B_* is the base position, *z_0_* is the amplitude of base vibration, *ω* is the vibration frequency, and *t* is time.

A KBP 206 rectifying bridge was connected to the piezo film’s output. Rectification must be included in experiments that are concerned with the final power output of harvesting as accounts for up to 50% of energy losses. The DC output was connected to a 100 μF/16 V capacitor. A 50 mW industrial 532 nm green laser line module was used to monitor the piezo film’s tip deflection and the cone speaker’s vibration amplitude. The laser highlighted the moving beam’s projection, which was captured with long-exposure photography with a NIKON D3400 camera. Measurements of the film’s tip deflection and the base’s amplitude were made with an image processing tool in Matlab/Octave.

Voltage measurements at the output of the rectifying bridge were recorded with a 16-bit 400 kSa/s data acquisition board (USB 6212, National Instruments). This was used in conjunction with a voltage divider circuit, whenever the output voltage exceeded 10 V. The test matrix of the experiments is presented in Table 3. The measurements were performed in three main phases. Initially, the excitation was exclusively from the base in the frequency range of 20–70 Hz. In this phase, the voltage output of the energy harvester to the capacitor was measured (◊). Additionally, long exposure photos of the laser sheet’s projection in a vertical plane along the main axis of the transducer (□) were taken and processed. In the second phase, the excitation was solely aerodynamic. In this phase, the same types of measurement were performed with the addition of mean flow velocity measurements (O). Next, the third phase of measurements was performed by repeating the first series of measurements 10 more times, with added aerodynamic excitations in the range of 1320 to 2640 rpm fan speed. In summary, 110 combinations of the 2 excitation modes were added.

## 3. Results and Discussion

This section presents the results of voltage measurements performed at the output of the rectifying bridge.

### 3.1. Capacitor Charging Curves

**Figure 6:**
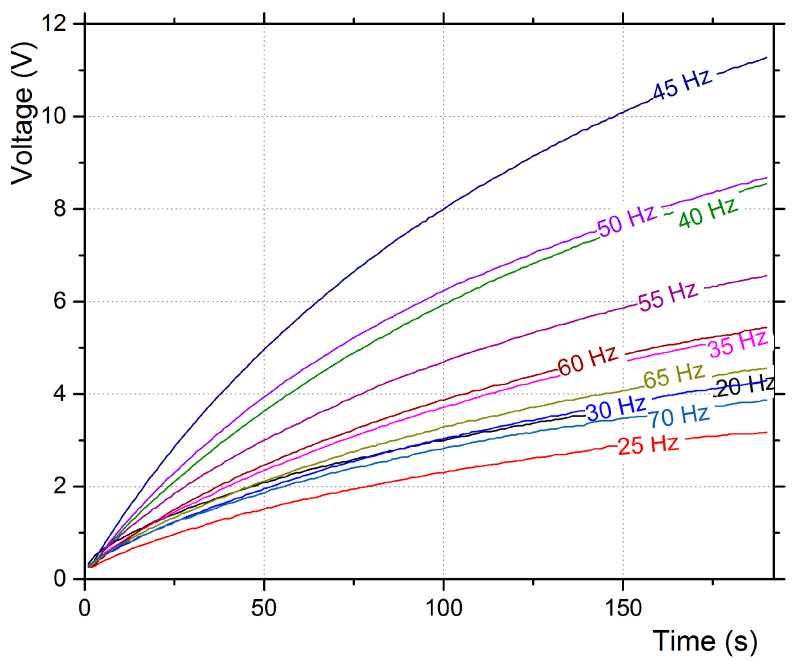
Voltage output to the 16 V/100 μF capacitor for base excitation only in various frequencies.

**Figure 7:**
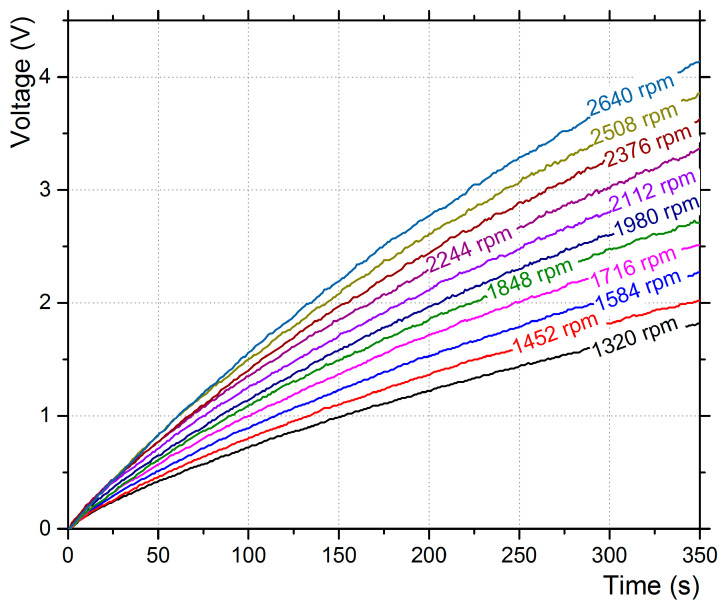
Voltage output to the 16 V/100 μF capacitor for aerodynamic excitation with various fan speeds.

The capacitor’s charging curves for a duration of 190 s are presented in Figure 6, for base excitation only, and Figure 7 for aerodynamic excitation for a duration of 350 s. In the case of base excitation, the piezo film’s oscillation frequency was the same as the base frequency.

The highest power harvested was at 45 Hz, and the second was higher at 50 Hz. The capacitor (16 V/100 μF) was charged up to 10.87 V after 175 s with 45 Hz excitation, which gives a power output of 33.4 μW. The average charging rate, in this case, is 10.87 V × 100 μF/175 = 6.21 μA. It should be noted that the highest power extracted was observed neither at the highest base vibration amplitude (30 Hz) nor at the base frequency closer to the first linear eigenfrequency of the piezoelectric transducer (58 Hz), but in the frequency range of 40–55 Hz. The same transducer produced a voltage rms that maximized when the base excitation frequency was the closest to its eigenfrequency, as presented in a previous study [13].

The capacitor’s charging curves for the aeroelastic excitation presented a different behavior with characteristic fluctuations in the curves (Figure 7). The capacitor was charged up to 4.2 V after 350 s with 2640 rpm fan speed, which gives an average power output of 2 μW. The increase of the mean flow velocity increased the power output in the measurement range.

**Figure 8:**
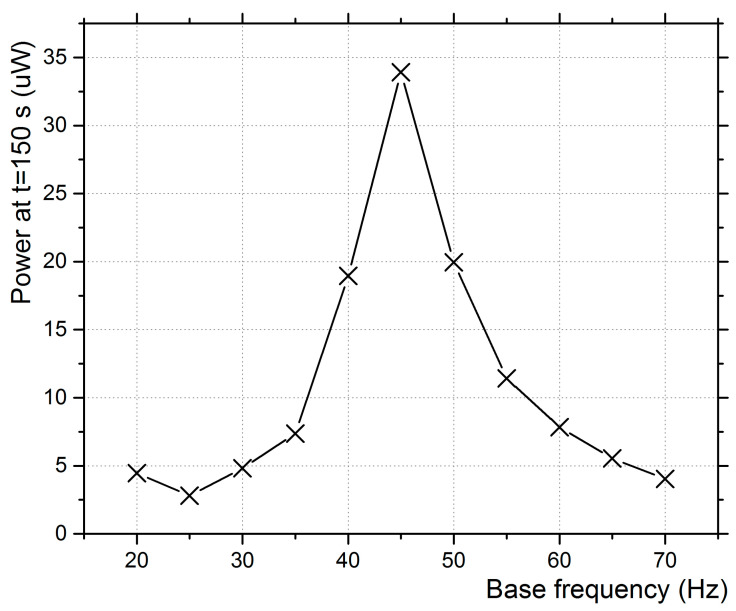
Power stored after 150 s vs. base frequency (only base excitation).

**Figure 9:**
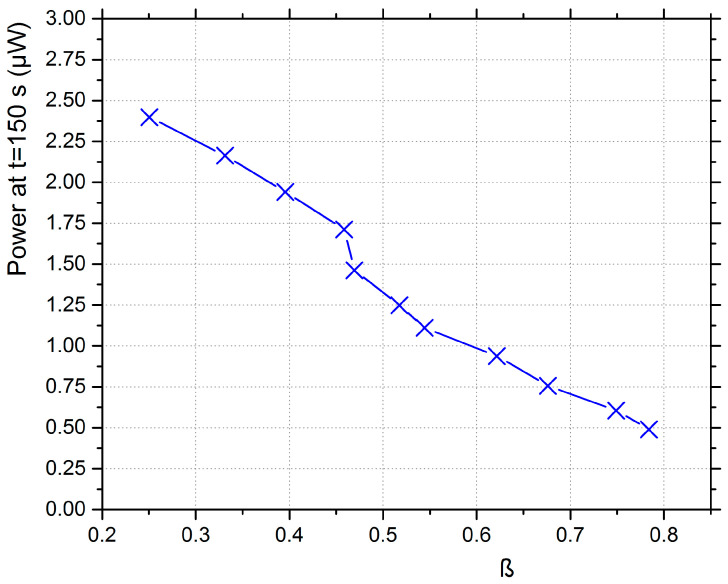
Power stored after 150 s vs. dimensionless bending stiffness (only aerodynamic excitation).

The harvested power after 150 s of excitation in exclusive base and exclusive aerodynamic excitation is presented in Figure 8 and Figure 9.

It is observed that the peak power output is at 45 Hz while the linear eigenfrequency of the piezoelectric transducer was measured to be 58 Hz. The forced excitation of the film’s base induces an eigenfrequency shift of about 13 Hz for the system. The eigenfrequency shift to lower values is due to the large deflections of the piezoelectric film, which lead to a nonlinear behavior [14,40]. According to the results of Elvin [41], who employed a generalized nonlinear coupled finite element circuit simulation approach to study the performance of energy harvesters subjected to large deflections, nonlinear dynamic effects become significant when the transverse tip deflection exceeds 35% of the beam length. Large deflections both shift the resonant frequency and increase damping and can thus cause a significant reduction in output voltage when compared with small-deflection linear theory. In the experiments of Figure 8 with base excitation, the highest power outputs are extracted at 35–55 Hz of base excitation.

The power output of the harvester is strongly dependent on the impedance of the load [42]. For the combined excitation point of 40 Hz and 1452 rpm, a capacitance study was carried out to find the optimal capacitance. Specifically, various capacitors in the range between 44 μF/16 V and 1100 μF/16 V were tested in the same excitation point (2376 rpm—35 Hz) in order to find the optimal capacitance. The capacitive load, which is a more realistic approximation of the actual electric load in PEH applications according to [43], was preferred in these tests to produce a level ground for comparison. The maximum power was harvested with the 100 μF/16 V capacitor. A capacitance of 44 to 400 μF was beneficial for the harvested power output as seen in Table 4.

As a final step, following the study of the two independent excitations acting on the piezoelectric film, measurements were performed with a combined excitation acting on the plate. In this case, the exciting force from pressure fluctuations, the sinusoidal force of the cone speaker, and the plate’s bending rigidity interact and cause the plate’s vibration. The power output was calculated as the energy stored in the capacitor from the relation

(9) $$P_{stored}=\frac{1}{2}C\left(V_{t2}^{2}-V_{t1}^{2}\right)/\Delta t$$

where $P_{stored}$ [μW] is the average stored power and C the capacitance [F].

**Figure 10:**
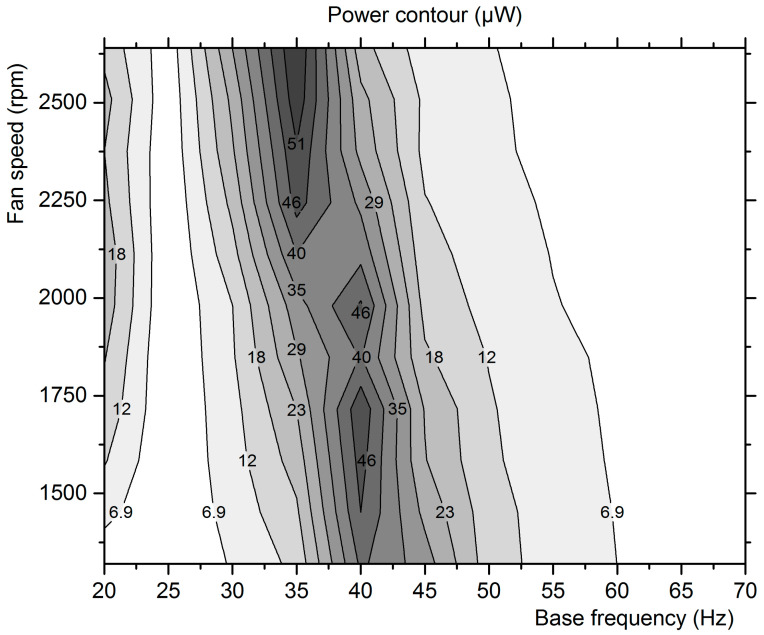
Contour map of mean power output after 150 s for fan and base frequency combinations.

The average power stored at the capacitor during the first 150 s, in all tested flow-induced and base excitation combinations of Table 3, is mapped in the contour diagram of Figure 10. The highest power output points are detected in two regions: around 40 Hz for a fan speed of 1400 to 2000 rpm and at 35 Hz for a fan speed of 2200 to 2600 rpm. The increase of the mean flow velocity induces large deflections on the film and makes the system’s eigenfrequency shift to a lower frequency. This is the effect of exciting higher modes of the PEH that induce amplitude modulation, as theoretically predicted by Bibo et al. [23]. In their study with combined excitation of a PEH, these researchers found that as the wind speed increases, the voltage–frequency response curves shift toward lower frequencies and higher output voltages are realized. Further, they predicted that below the flutter speed, the response of the harvester remains periodic with the airflow amplifying the influence of the base excitation by reducing the effective stiffness of the system and increasing RMS output. Beyond the flutter speed, Bibo et al. [23] observed two distinct regions: (i) those with small base excitation or when excitation frequency is far from the self-sustained oscillations frequency induced by the flutter instability. In this case, the response is two-period quasiperiodic with amplitude modulation, which reduces RMS output; (ii) regions where the amplitude of excitation is large enough to quench the quasiperiodic response by causing the two frequencies to lock into each other, with the response becoming periodic and the output power increasing again, exhibiting little dependence on the base excitation amplitude. The highest power harvested in these experiments is 56.6 μW, at maximum fan speed and 35 Hz of base excitation. It is observed that the constituent excitations produce significantly lower power outputs than their combination. This is valid also for the absolute maximum performance points, clearly demonstrated by the fact that the highest power harvested is 56.6 μW at combined excitation mode, compared to a maximum of 33.4 μW with base excitation only and about 2 μW maximum output with aeroelastic excitation alone.

**Figure 11:**
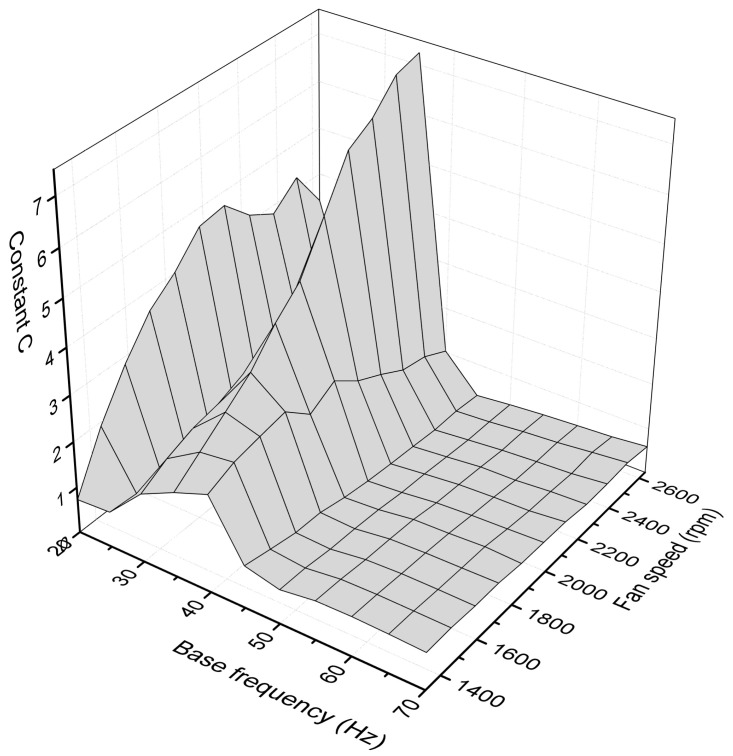
Surface map of constant c.

The highest power harvested was 56.6 μW at maximum fan speed and 35 Hz of base excitation. It was observed that the constituent excitations produce much lower power outputs than their combination. To investigate the measurement points where the combination is beneficial to the power output, the constant c is introduced as follows:

(10) $$c=\frac{P_{comb}}{P_{base}+P_{flow}}$$

where $P_{comb}$ is the power output of the combined excitation, $P_{base}$ is the power output if the base excitation acted alone on the piezo film, and $P_{flow}$ is the power output if the flow excitation acted alone. This constant is mapped in Figure 11.

The degree of synergy between the two excitation modes depends on the base frequency, as in some frequencies the flow excitation seems to be beneficial, while in others, it is disadvantageous for the power output. Specifically, the combination is beneficial in the base excitations of 20, 30, 35, and 40 Hz.

### 3.2. Laser Sheet Projections

**Figure 12:**
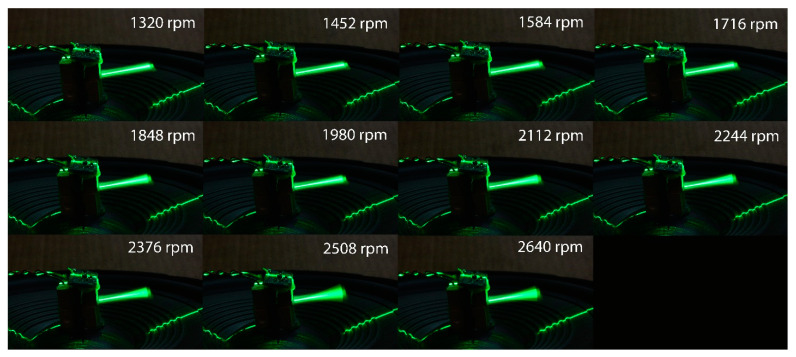
Laser projections of the piezo film with only flow excitation in various fan speeds.

**Figure 13:**
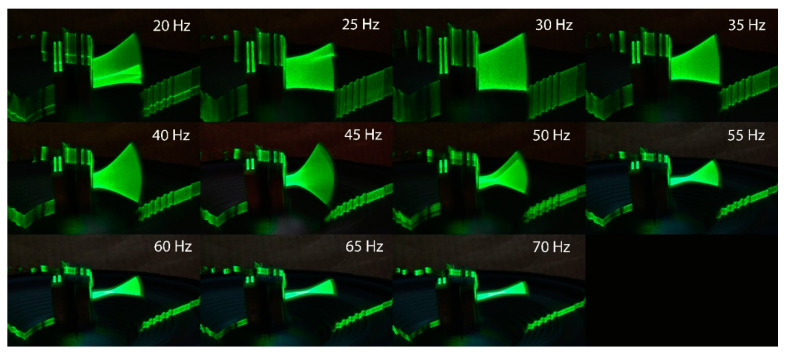
Laser projection of the piezo film with only base excitation in various frequencies.

**Figure 14:**
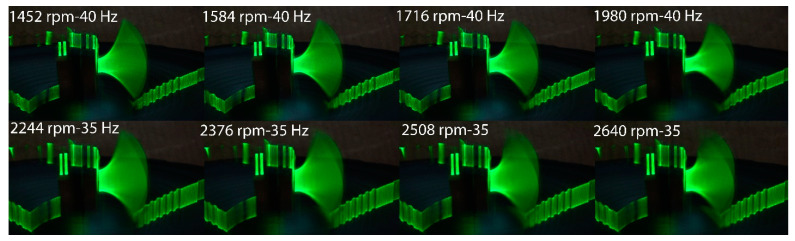
Laser projection of the 4 points with maximum harvested power.

A line-type laser was used to highlight the piezo film’s movement. The movement was captured by photographing the subject with 1 s exposure. The photographs revealed the different eigenmodes of the piezo film for each vibration frequency. The resulting projections of only fan excitation, only base excitation, and the projection for the maximum power output points are presented in Figure 12, Figure 13 and Figure 14.

The piezo film presented small deflections for the flow speed range tested in these experiments. This is due to a low value of dimensionless bending stiffness [21] leaving the film’s vibration outside the flapping range. The vibration resulted in low power outputs. The cut-in flow velocity was about 10 m/s.

It is observed from Figure 13 that the base excitation in the range of 20–55 Hz induced large deflections of the piezo film. This is a source of geometric nonlinearity that cannot be described with classical beam theory [44], while efforts have recently been made to investigate the effects of nonlinearities in PEH [23,25,45]. It is observed that at the points with large deflections, there is a shift of the system’s natural frequency to a lower value. In base frequencies, greater than 50 Hz a phase lag of 180 degrees is observed between the film and the base’s vibration. The lag results in a lower curvature in the top and bottom positions of the vibration. In the frequencies that the lag exists, the flow excitation is disadvantageous to the power output. The phase difference between the excitation and the system response has been observed in experiments by other researchers, with an added tip mass [27]. In low-frequency conditions, the response kept pace with the base excitation. In high-frequency conditions, the response lagged behind the excitation with about 180°.

**Figure 15:**
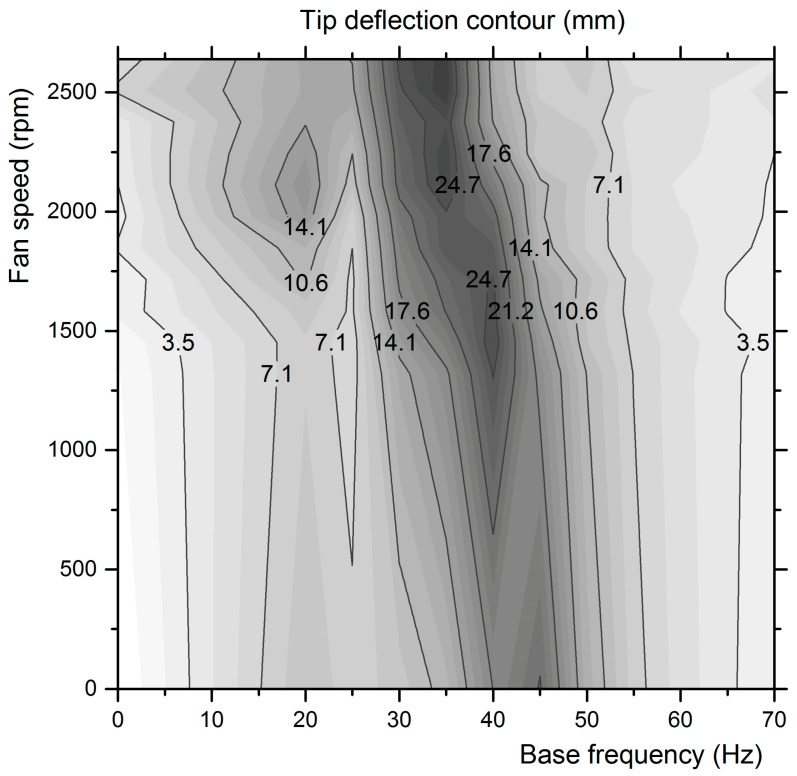
Tip deflection map in different combinations of base and fan frequencies.

In Figure 14, the projection of combinations of excitation with the highest power outputs is presented. Obviously, these excitation combinations also produce the highest tip deflections and curvatures on the film during its oscillation extrema, resulting in high stresses on the film and thus a high charge output per vibration period. A devised curvature increase can thus produce a higher power output in vibration frequencies with appropriate mode shapes. This can be used to broaden the bandwidth of harvesters. The tip deflection of the piezo film is presented for various base frequency and fan speed combinations in Figure 15. This map presents similarities to the map of the power output of Figure 10 regarding the areas with maximum points.

The maximum tip deflection of the piezo film, along with the exact shape of the elastic line and its curvature variation along the beam’s length, can be measured with high accuracy by applying image processing in MATLAB. The maximum tip deflection was used for the strain energy computations described in the next section.

## 4. Strain Energy Computations

For each oscillation stroke of the transducer, starting from its neutral position to its lower position, an amount of electric charge is displaced and may be transferred through the transducer’s terminals to charge an external capacitor after being rectified. An approximately equal and opposite charge is produced upon the return of the transducer from its lower extent toward the neutral. In summary, provided that a perfect rectifier diode circuit is connected to the transducer’s terminals, a total of four Q would charge the externally connected capacitor for every full period of oscillation. It is assumed that the power output to the external capacitor would amount to four Q f, where f is the frequency of oscillation, and Q is the electric charge displaced during bending of the thin beam from its neutral to its maximum bending position. On an energy basis, the charge output energy may be assumed to be produced by a transformation of the total strain energy of the piezo-beam as it goes from neutral to full flexure position. To maximize the charge output of the piezo film, it is necessary to maximize the product: (frequency of oscillation) × (strain energy at the point of maximum tip deflection). The product is expected to be maximized during resonance. In the absence of damping, this will be the natural frequency of the beam. A beam made of elastic material that obeys Hooke’s law is known to deform in such a way that the curvature *K* of the elastic curve is proportional to the bending moment *M*.

(11) $$K=\frac{y^{\prime \prime }}{{\left[1+{\left(y^{\prime }\right)}^{2}\right]}^{\frac{3}{2}}}=\frac{M}{EI}$$

where *E* is Young’s modulus, *I* is the moment of inertia of the cross section of the beam about a horizontal line passing through the centroid of the section and lying in the plane of the cross section, and *y* is the ordinate of the elastic curve. This is the Bernoulli–Euler law. The elastic strain energy *E_S_* of the piezo film is defined by integration over its length as follows:

(12) $$E_{S}\left(t\right)=\int_{0}^{L}\frac{1}{2}BK{\left(s,t\right)}^{2}ds$$

where the beam curvature is a function of both the position along its length and time, and *B* is the flexural rigidity of the piezo film,

(13) $$B=\frac{Eh^{3}}{12\left(1-v^{2}\right)}$$

where *h* is the film’s thickness, *E* is Young’s modulus 2.4 × 10^9^ N/m^2^, and *ν* is Poisson’s ratio ν = 0.38 for a polycarbonate film. Now, it can be assumed as a first approximation that the electrical energy stored in the capacitor at the output terminals of the piezoelectric transducer during one-fourth of its period of oscillation will be proportional to its elastic strain energy at its maximum tip deflection position as follows:

(14) $$E_{cap}=\eta \;E_{S}\left(t_{max}\right)=\eta \int_{0}^{L}\frac{1}{2}BK{\left(s,t_{max}\right)}^{2}ds$$

where *η* is the energy conversion efficiency, $t_{max}$ is the time corresponding to an oscillation extremum (maximum tip deflection points), and the value of *B* in our specific case of interest is

(15) $$B=\frac{Ewh^{3}}{12\left(1-v^{2}\right)}=\frac{2.4\;\times 10^{9}\;\left(0.016\right){\left(28\;\times 10^{-6}\right)}^{3}}{12\left(1-0.38^{2}\right)}=8.21\times \;10^{-8}\;{\mathrm{Nm}}^{2}$$

**Figure 16:**
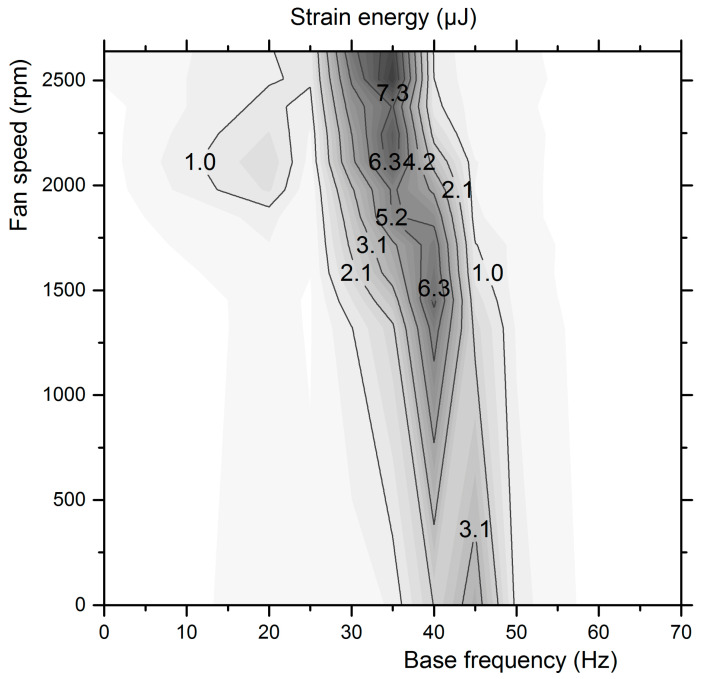
Transducer maximum strain energy map in combined base and aerodynamic excitation frequencies.

The instants with maximum tip deflection (upper and lower oscillation extrema positions) were captured with laser sheet lighting, as demonstrated in Figure 14. The elastic curve was digitized from each photograph and approximated by polynomial regression. Thus, the integral of Equation (13) can be computed. In this way, the strain energy of the piezo film at its oscillation extrema is calculated as a function of its maximum tip deflection points, mapped in Figure 15. To speed up the computation, the results can be fitted by a third degree polynomial, which correlates the strain energy at the beam’s oscillation extrema to the maximum tip deflection as follows:

(16) $$E_{S}\left(t_{max}\right)=a_{3}\;x^{3}+a_{2}\;x^{2}+a_{1}\;x\;$$

where *x* is the maximum tip deflection and the coefficients α_1_, α_2_, α_3_ take the values 7 × 10^−8^, 6 × 10^−9^, and 5 × 10^−10^, respectively for the specific piezoelectric film. Thus, the tip deflection of the piezo film that has been accurately measured for various base frequency and fan speed combinations (Figure 15) is transformed to the strain energy map, as presented in Figure 16.

**Figure 17:**
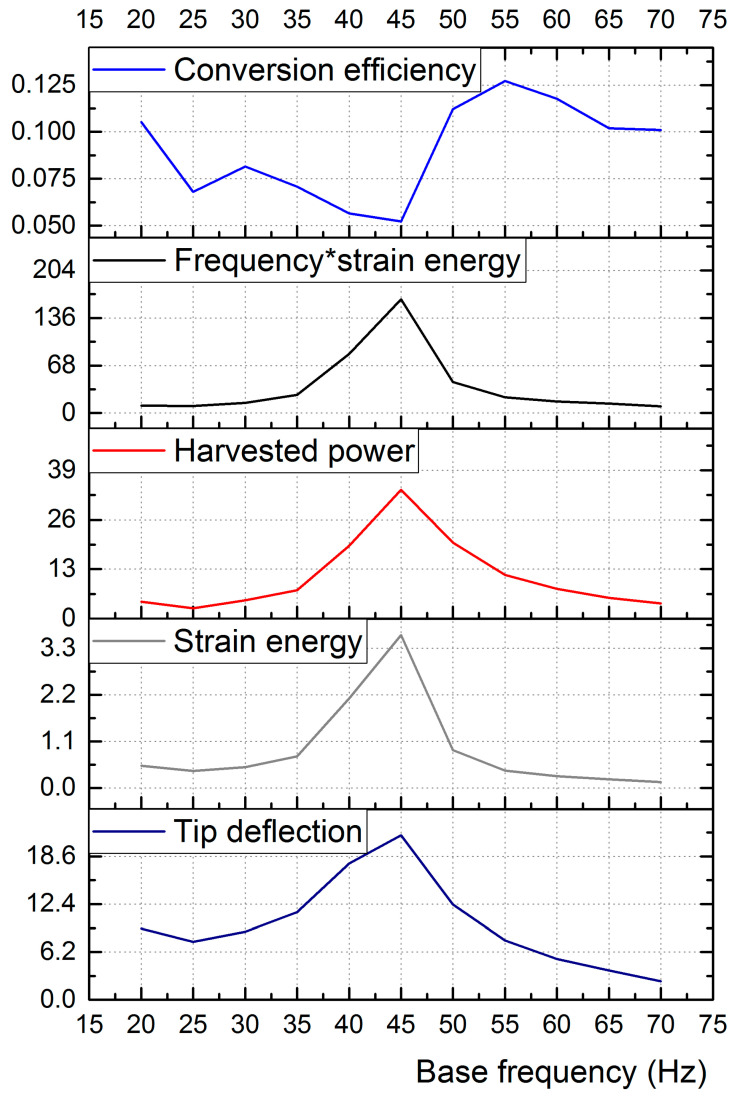
Correlation of the harvested power to the product of (oscillation frequency) × (strain energy) at the oscillation extrema (base excitation only).

This procedure allows a better insight into the correlation of the harvester’s output with the strain energy calculated based on the beam’s curvature at its oscillation extrema. According to the results presented in Figure 17, for base vibration only (base excitation frequency in the range of 20–70 Hz), the harvested power may be predicted based on the strain energy at maximum tip deflection with acceptable accuracy.

**Figure 18:**
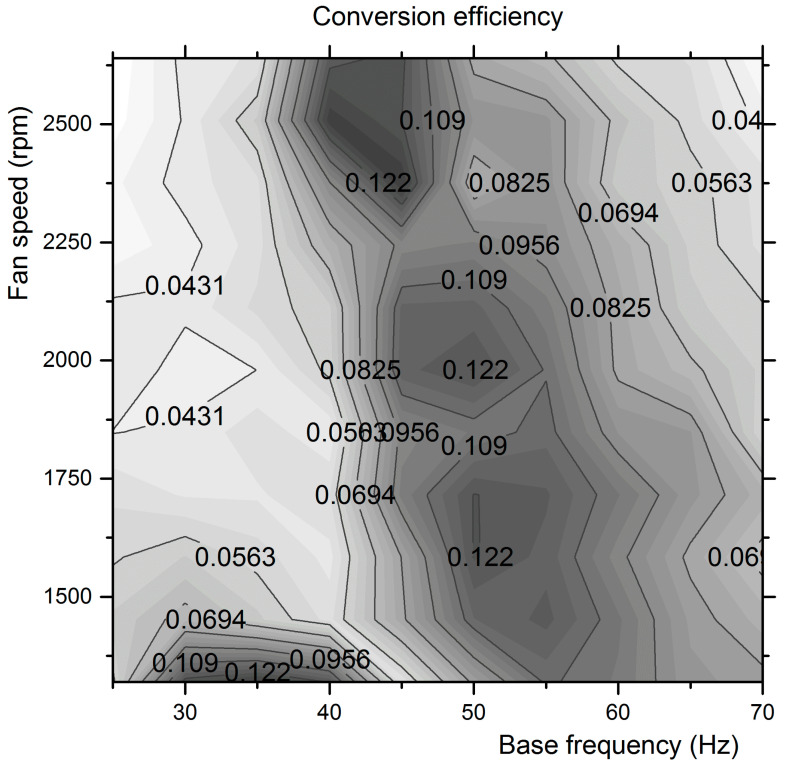
Mapping the energy efficiency for harvested power estimation in the combined base and aerodynamic excitation space.

The harvested power curve presents a strong similarity to the curve of the (base oscillation frequency) × (maximum strain energy) product, also presented in this Figure. Each period of oscillation comprises four strain energy change steps (i.e., from upper extremum to neutral line, from neutral to lower extremum, and the opposite two changes), and the conversion efficiency is defined by dividing the harvested power to the quadruple of the product (frequency × strain energy). The conversion efficiency calculated lies in the range of 0.05–0.13. Using this definition, a map of the harvester’s conversion efficiency for all combinations of excitation measured was created and presented in Figure 18.

According to the map, the conversion efficiency values range from 0.03 to 0.14 in the full space of combinations of 25–70 Hz base vibration frequency and fan speeds from 1320 to 2640 rpm. The highest values are observed at 45 Hz base frequency and maximum fan speed. This is the range with the highest tip deflections and highest harvested power measured. The reported efficiency values generally agree with the range reported by several researchers [25,27]. On the other hand, as already discussed in the introduction, several different definitions exist in the literature, and this partially explains the fact that the efficiency values reported may differ by orders of magnitude. Since the energy flow in an energy-harvesting system comprises the excitation source, the PEH, and the rectification circuit up to the storage capacitor or battery, if one defines the input energy as the energy supplied by the excitation source to the PEH structure before it is converted to strain energy, then the resulting efficiency would seem very low. For example, in the specific test rig employed in this study, the cone speaker would receive a few Watts from the amplifiers, and the flow exiting the fan would have a power of a few Watts (computed as the product of volume flowrate times backpressure). The fact that we defined the PEH efficiency, not as overall energy efficiency in the above sense, but only to cover the transformation of the transducer’s strain energy to electric energy, creates a level ground for comparison of different transducers by excluding the effects of the design and implementation of their specific testing facilities. As demonstrated by the measurements and processing reported in this paper, the specific test rigs and processing codes allow for fast screening and performance mapping of different types of PEHs, to focus on optimum energy-harvesting efficiency and assessment of modified design versions to fit specific energy-harvesting applications.

## 5. Conclusions

Experiments with a piezoelectric energy-harvesting system in combined excitation were performed. The PVDF film was subjected to aerodynamic and base excitation separately and in various combinations. The harvested power was mapped along with the maximum tip deflection of the piezo film and a process of optimally combining the two excitation sources for maximum power harvesting was demonstrated. The highest harvested power was 56.6 μW in a combined excitation point, which is, to the authors’ knowledge, the highest power output reported with the specific piezoelectric transducer. By subjecting the piezoelectric film to combined excitation, significantly more power can be harvested than with the two excitations acting separately. The combination of aeroelastic and sinusoidal base excitation was found to be beneficial in some base excitation frequencies because of the collaboration of the two types of excitations in increasing the film’s curvature during oscillation extrema, while in other frequencies, it was disadvantageous due to 180° phase lag between the piezo film and the cone’s vibration. Eigenfrequency shifts were observed that are attributed to the large beam deflections caused by the forced excitations of the harvester and, further, by the complex interaction with the flow excitation. The combinations of excitation that produced the highest beam curvature showed the highest power outputs and efficiencies. The curvature of the mode shapes was found to be an accurate predictor of the harvested power output. Although the vibration of PEHs is usually modeled as linear, large deflections that induce geometric nonlinearities are common in applications. Elastic films with large deformations signify a stronger interaction of the film with the excitation source and a higher capability to harvest energy. The test rigs and processing algorithms applied in this paper allow a fast assessment and performance mapping of different types of PEHs, toward attaining optimum energy-harvesting efficiency.

## Figures and Tables

**Table 1:** LDT1-028K film characteristics [36].

Commercial Name	Piezoelectric Material	Total Length	Width	Thickness	Substrate Material	Capacitance [nF]	Measured Eigen-Frequency [13]
LDT1-028K	PVDF	41 mm	16 mm	28 μm	Mylar	1.38	58 Hz

**Table 2:** Piezoelectric charge coefficients for PVDF [37].

*d*_15_ [pC/N]	*d*_24_ [pC/N]	*d*_31_ [pC/N]	*d*_32_ [pC/N]	*d*_33_ [pC/N]
−27	−23	21	1.5	−32.5

**Table 3:** Test matrix with all the combinations of base excitation frequency (horizontal direction) with aerodynamic excitation (fan speed in the vertical direction). The specific symbols denote: ◊ capacitor voltage measurements, □ laser projection measurements, O mean flow velocity measurements.

	0 Hz	20 Hz	25 Hz	30 Hz	35 Hz	40 Hz	45 Hz	50 Hz	55 Hz	60 Hz	65 Hz	70 Hz
0 rpm	O□	◊□	◊□	◊□	◊□	◊□	◊□	◊□	◊□	◊□	◊□	◊□
1320 rpm	O◊□	◊□	◊□	◊□	◊□	◊□	◊□	◊□	◊□	◊□	◊□	◊□
1452 rpm	O◊□	◊□	◊□	◊□	◊□	◊□	◊□	◊□	◊□	◊□	◊□	◊□
1584 rpm	O◊□	◊□	◊□	◊□	◊□	◊□	◊□	◊□	◊□	◊□	◊□	◊□
1716 rpm	O◊□	◊□	◊□	◊□	◊□	◊□	◊□	◊□	◊□	◊□	◊□	◊□
1848 rpm	O◊□	◊□	◊□	◊□	◊□	◊□	◊□	◊□	◊□	◊□	◊□	◊□
1980 rpm	O◊□	◊□	◊□	◊□	◊□	◊□	◊□	◊□	◊□	◊□	◊□	◊□
2112 rpm	O◊□	◊□	◊□	◊□	◊□	◊□	◊□	◊□	◊□	◊□	◊□	◊□
2244 rpm	O◊□	◊□	◊□	◊□	◊□	◊□	◊□	◊□	◊□	◊□	◊□	◊□
2376 rpm	O◊□	◊□	◊□	◊□	◊□	◊□	◊□	◊□	◊□	◊□	◊□	◊□
2508 rpm	O◊□	◊□	◊□	◊□	◊□	◊□	◊□	◊□	◊□	◊□	◊□	◊□
2640 rpm	O◊□	◊□	◊□	◊□	◊□	◊□	◊□	◊□	◊□	◊□	◊□	◊□

**Table 4:** LDT1-028K film characteristics [36].

Capacitance (μF)	44	100	188	408	878	1100
Harvested power (μW)	26.1	45.6	38.1	29.9	20.3	9.7
